# 3-[2-(9-Ethyl-9*H*-carbazol-3-yl)-6-methyl-3-quinol­yl]propan-1-ol

**DOI:** 10.1107/S1600536808042153

**Published:** 2008-12-17

**Authors:** S. Murugavel, S. Ranjith, A. SubbiahPandi, G. Periyasami, R. Raghunathan

**Affiliations:** a Department of Physics, Thanthai Periyar Government Institute of Technology, Vellore 632 002, India; b Department of Physics, Presidency College (Autonomous), Chennai 600 005, India; c Department of Organic Chemistry, University of Madras, Guindy Campus, Chennai 600 025, India

## Abstract

In the title compound, C_27_H_26_N_2_O, the mean planes through the carbazole and quinoline ring systems form a dihedral angle of 67.23 (5)°. Mol­ecules are linked into cyclic centrosymmetric dimers by O—H⋯N hydrogen bonds, and C—H⋯π inter­actions with the pyridine ring of the quinoline ring system as an acceptor. The dimers are linked through C—H⋯O hydrogen bonds.

## Related literature

For the biological activity and applications of carbazole derivatives, see: Itoigawa *et al.* (2000 ▶); Tachibana *et al.* (2001 ▶); Ramsewak *et al.* (1999 ▶); Friend *et al.* (1999 ▶); Diaz *et al.* (2002 ▶); Zhang *et al.* (2004 ▶). For the biological properties of quinoline derivatives, see: Cunico *et al.* (2006 ▶); Hartline *et al.* (2005 ▶). For related structures, see: Murugavel *et al.* (2008 ▶); Chakkaravarthi *et al.* (2008 ▶).
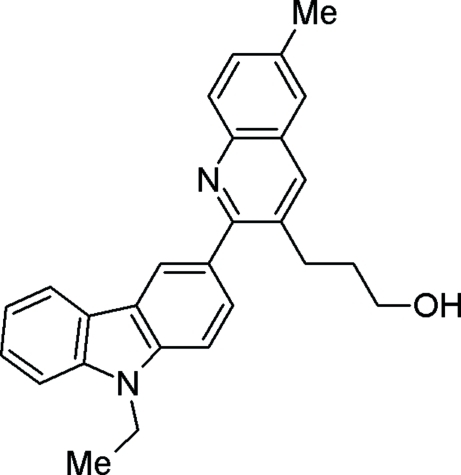


## Experimental

### 

#### Crystal data

C_27_H_26_N_2_O
*M* *_r_* = 394.50
Monoclinic, 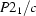

*a* = 13.6417 (5) Å
*b* = 9.8599 (3) Å
*c* = 16.3208 (5) Å
β = 109.658 (2)°
*V* = 2067.30 (12) Å^3^
*Z* = 4
Mo *K*α radiation
μ = 0.08 mm^−1^
*T* = 293 (2) K
0.21 × 0.19 × 0.17 mm

#### Data collection

Bruker APEXII CCD area-detector diffractometer
Absorption correction: multi-scan (*SADABS*; Sheldrick, 1996 ▶) *T*_min_ = 0.984, *T*_max_ = 0.987
27360 measured reflections
6459 independent reflections
3912 reflections with *I* > 2σ(*I*)
*R*_int_ = 0.033

#### Refinement

*R*[*F* ^2^ > 2σ(*F* ^2^)] = 0.065
*wR*(*F* ^2^) = 0.228
*S* = 1.02
6459 reflections
316 parameters
H atoms treated by a mixture of independent and constrained refinement
Δρ_max_ = 0.67 e Å^−3^
Δρ_min_ = −0.39 e Å^−3^

### 

Data collection: *APEX2* (Bruker, 2004 ▶); cell refinement: *SAINT* (Bruker, 2004 ▶); data reduction: *SAINT*; program(s) used to solve structure: *SHELXS97* (Sheldrick, 2008 ▶); program(s) used to refine structure: *SHELXL97* (Sheldrick, 2008 ▶); molecular graphics: *ORTEP-3* (Farrugia, 1997 ▶); software used to prepare material for publication: *SHELXL97* and *PLATON* (Spek, 2003 ▶).

## Supplementary Material

Crystal structure: contains datablocks global, I. DOI: 10.1107/S1600536808042153/ci2733sup1.cif

Structure factors: contains datablocks I. DOI: 10.1107/S1600536808042153/ci2733Isup2.hkl

Additional supplementary materials:  crystallographic information; 3D view; checkCIF report

## Figures and Tables

**Table 1:** Hydrogen-bond geometry (Å, °)

*D*—H⋯*A*	*D*—H	H⋯*A*	*D*⋯*A*	*D*—H⋯*A*
O1—H1⋯N2^i^	0.82	2.18	2.987 (2)	166
C17—H17⋯O1^ii^	0.97 (2)	2.38 (2)	3.334 (3)	171 (2)
C10—H10⋯*Cg*1^i^	0.96 (3)	2.76 (2)	3.559 (2)	141 (2)

Symmetry codes: (i) 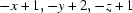; (ii) 
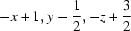
. *Cg*1 is the centroid of the N2/C15–C19 ring.

**Fig. 1.**
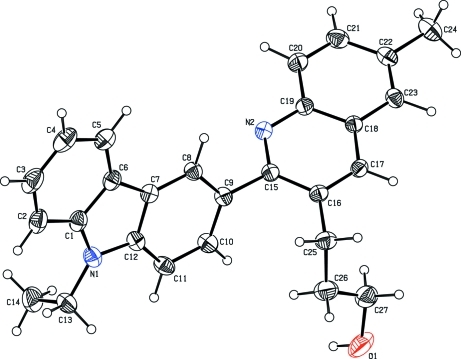
The molecular structure of the title compound showing the atomic labelling scheme. Displacement ellipsoids are drawn at the 30% probability level and H atoms are shown as small spheres of arbitory radii.

**Fig. 2.**
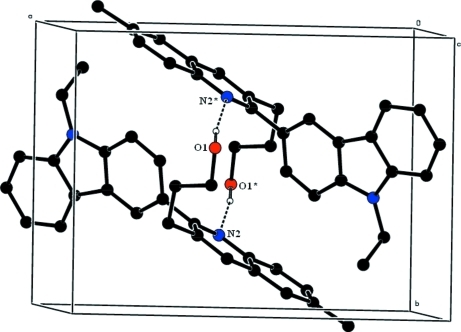
A view of the centrosymmetric R22(16) dimer in the crystal structure of the title compound. Atoms marked with an asterisk (*) are at the symmetry position (1 - x, 2 - y, 1 - z). H atoms not involved in the hydrogen bonds (dashed lines) have been omitted.

## supplementary crystallographic information

### Comment

Carbazole and its derivatives have become quite attractive compounds owing to their applications in pharmacy and molecular electronics. It has been reported that carbazole derivatives possess various biological activities, such as antitumor (Itoigawa *et al.*, 2000), antioxidative (Tachibana *et al.*, 2001), anti-inflammatory and antimutagenic (Ramsewak *et al.*, 1999) activities. Carbazole derivatives also exhibit electroactivity and luminescence properties and are considered to be potential candidates for electronics such as colour displays, organic semiconductor lasers and solar cells (Friend *et al.*, 1999). These compounds are thermally and photochemically stable, which makes them useful materials for technological applications. For instance, the carbazole ring is easily funtionalized and covalently linked to other molecules (Diaz *et al.*, 2002). This enables its use as a convenient building block for the design and synthesis of molecular glasses, which are widely studied as components of electroactive and photoactive materials (Zhang *et al.*, 2004). Quinoline derivatives are known to possess a variety of biological properties such as antimalarial and antiviral activity (Cunico *et al.*, 2006; Hartline *et al.*, 2005). Against this background, and in order to obtain detailed information on molecular conformations in the solid state, an X-ray study of the title compound was carried out.

The carbazole and quinoline ring systems are individually planar, with maximum deviations of 0.077 (1) and 0.034 (2) Å for atoms C9 and C22, respectively. The sum of the bond angles around N1 (351.7°) of the pyrrole ring is in accordance with *sp*^2^ hybridization. The N1—C1 [1.372 (3) Å] and N1—C12 [1.382 (2) Å] bond lengths are normal and are comparable with the corresponding values observed in a related structure (Chakkaravarthi *et al.*, 2008; Murugavel *et al.*, 2008). The mean planes through the carbazole and quinoline ring systems form a dihedral angle of 67.23 (5)°.

The molecules at (x, y, z) and (1-x, 2-y, 1-z) are linked by O1—H1···N2 hydrogen bonds into a cyclic centrosymmetric *R*_2_^2^(16) dimer (Fig. 2). Within the dimer, C10–H10···π interactions with the pyridine ring of the quinoline ring system as an acceptor are observed. Atom C17 in the molecule (*x*, *y*, *z*) donate one proton to atom O1 of the molecule at (1 - *x*, -1/2 + *y*, 3/2 - *z*) forming a C(7) chain along the [010].

### Experimental

InCl_3_ (20 mol%) was added to a mixture of 3,4-dihydro-2*H*-pyran (1 mmol) and *N*-[(9-Ethyl-9*H*-carbazol-6-yl)methylene]-4-methylbenzenamine (1 mmol) in dry acetonitrile (10 ml) and the reaction mixture was refluxed until the completion of the reaction as indicated by TLC. The reaction mixture was then quenched with water, extracted with excess of ethyl acetate. The organic layer was washed with water and dried over anhydrous MgSO_4_. The solvent was evaporated *in vacuo* and the crude product was chromatographed on silica gel (ethylacetate-hexane, 2:8) to afford quinoline propanol. The pure compound was crystallized from ethylacetate-hexane 1:9). Single crystals suitable for X-ray diffraction were obtained by slow evaporation of a ethylacetate solution at room temperature.

### Refinement

Ring H atoms were located in a difference map and refined freely. All other H atoms were positioned geometrically (O-H = 0.82 Å and C-H = 0.96 or 0.97 Å) and allowed to ride on their parent atoms, with *U*_iso_(H) = 1.5*U*_eq_(carrier) for methyl and hydroxy H atoms and 1.2*U*_eq_(C) for other H atoms. The highest residual density peak is located 0.95 Å from atom H26A and the deepest hole is located 0.58 Å from atom C26.

### Crystal data

**Table:**

C_27_H_26_N_2_O	*F*(000) = 840
*M**_r_* = 394.50	*D*_x_ = 1.268 Mg m^−^^3^
Monoclinic, *P*2_1_/*c*	Mo *K*α radiation, λ = 0.71073 Å
Hall symbol: -P 2ybc	Cell parameters from 6459 reflections
*a* = 13.6417 (5) Å	θ = 1.6–30.8°
*b* = 9.8599 (3) Å	µ = 0.08 mm^−^^1^
*c* = 16.3208 (5) Å	*T* = 293 K
β = 109.658 (2)°	Block, colourless
*V* = 2067.30 (12) Å^3^	0.21 × 0.19 × 0.17 mm
*Z* = 4	

### Data collection

**Table:**

Bruker APEXII CCD area-detector diffractometer	6459 independent reflections
Radiation source: fine-focus sealed tube	3912 reflections with *I* > 2σ(*I*)
graphite	*R*_int_ = 0.033
ω scans	θ_max_ = 30.8°, θ_min_ = 1.6°
Absorption correction: multi-scan (*SADABS*; Sheldrick, 1996)	*h* = −19→19
*T*_min_ = 0.984, *T*_max_ = 0.987	*k* = −14→13
27360 measured reflections	*l* = −23→23

### Refinement

**Table:**

Refinement on *F*^2^	Primary atom site location: structure-invariant direct methods
Least-squares matrix: full	Secondary atom site location: difference Fourier map
*R*[*F*^2^ > 2σ(*F*^2^)] = 0.065	Hydrogen site location: inferred from neighbouring sites
*wR*(*F*^2^) = 0.228	H atoms treated by a mixture of independent and constrained refinement
*S* = 1.02	*w* = 1/[σ^2^(*F*_o_^2^) + (0.1233*P*)^2^ + 0.4813*P*] where *P* = (*F*_o_^2^ + 2*F*_c_^2^)/3
6459 reflections	(Δ/σ)_max_ = 0.006
316 parameters	Δρ_max_ = 0.67 e Å^−^^3^
0 restraints	Δρ_min_ = −0.38 e Å^−^^3^

### Special details

**Table:**

Geometry. All e.s.d.'s (except the e.s.d. in the dihedral angle between two l.s. planes) are estimated using the full covariance matrix. The cell e.s.d.'s are taken into account individually in the estimation of e.s.d.'s in distances, angles and torsion angles; correlations between e.s.d.'s in cell parameters are only used when they are defined by crystal symmetry. An approximate (isotropic) treatment of cell e.s.d.'s is used for estimating e.s.d.'s involving l.s. planes.
Refinement. Refinement of *F*^2^ against ALL reflections. The weighted *R*-factor *wR* and goodness of fit *S* are based on *F*^2^, conventional *R*-factors *R* are based on *F*, with *F* set to zero for negative *F*^2^. The threshold expression of *F*^2^ > σ(*F*^2^) is used only for calculating *R*-factors(gt) *etc*. and is not relevant to the choice of reflections for refinement. *R*-factors based on *F*^2^ are statistically about twice as large as those based on *F*, and *R*- factors based on ALL data will be even larger.

### Fractional atomic coordinates and isotropic or equivalent isotropic displacement parameters (Å^2^)

**Table:**

	*x*	*y*	*z*	*U*_iso_*/*U*_eq_	
C1	0.93457 (15)	1.0043 (2)	0.38448 (12)	0.0457 (4)	
C2	1.02298 (17)	1.0150 (3)	0.36015 (14)	0.0593 (6)	
C3	1.0529 (2)	0.9032 (3)	0.32531 (16)	0.0702 (7)	
C4	0.9975 (2)	0.7822 (3)	0.31265 (16)	0.0682 (7)	
C5	0.90952 (18)	0.7707 (2)	0.33655 (14)	0.0545 (5)	
C6	0.87763 (14)	0.8820 (2)	0.37340 (11)	0.0435 (4)	
C7	0.79523 (13)	0.90547 (18)	0.40869 (11)	0.0392 (4)	
C8	0.71771 (14)	0.82534 (18)	0.42123 (12)	0.0404 (4)	
C9	0.65428 (13)	0.87827 (17)	0.46436 (12)	0.0393 (4)	
C10	0.66606 (15)	1.01400 (19)	0.49038 (14)	0.0455 (4)	
C11	0.74047 (15)	1.09668 (19)	0.47741 (14)	0.0471 (4)	
C12	0.80606 (14)	1.04043 (19)	0.43802 (12)	0.0417 (4)	
C13	0.92173 (18)	1.2385 (2)	0.44090 (15)	0.0562 (5)	
H13A	0.9950	1.2464	0.4479	0.067*	
H13B	0.9135	1.2646	0.4955	0.067*	
C14	0.8608 (2)	1.3349 (3)	0.37111 (17)	0.0729 (7)	
H14A	0.8850	1.4257	0.3870	0.109*	
H14B	0.7883	1.3291	0.3646	0.109*	
H14C	0.8701	1.3114	0.3171	0.109*	
C15	0.57097 (13)	0.79261 (17)	0.47778 (11)	0.0369 (4)	
C16	0.57191 (13)	0.75609 (17)	0.56270 (11)	0.0382 (4)	
C17	0.49310 (14)	0.67525 (18)	0.56791 (11)	0.0391 (4)	
C18	0.41231 (13)	0.63336 (16)	0.49268 (11)	0.0364 (3)	
C19	0.41835 (13)	0.67418 (17)	0.41187 (11)	0.0385 (4)	
C20	0.33782 (17)	0.6347 (2)	0.33508 (13)	0.0514 (5)	
C21	0.25614 (17)	0.5612 (2)	0.33960 (15)	0.0555 (5)	
C22	0.24826 (15)	0.52069 (19)	0.42029 (14)	0.0479 (4)	
C23	0.32661 (15)	0.55485 (18)	0.49506 (13)	0.0437 (4)	
C24	0.15449 (18)	0.4418 (2)	0.42195 (18)	0.0658 (6)	
H24A	0.1392	0.4643	0.4736	0.099*	
H24B	0.1686	0.3464	0.4217	0.099*	
H24C	0.0959	0.4643	0.3716	0.099*	
C25	0.65808 (16)	0.7989 (2)	0.64488 (13)	0.0507 (5)	
H25A	0.7222	0.8040	0.6317	0.061*	
H25B	0.6669	0.7274	0.6876	0.061*	
C26	0.6451 (2)	0.9318 (3)	0.68702 (16)	0.0670 (6)	
H26A	0.7073	0.9479	0.7368	0.080*	
H26B	0.6398	1.0046	0.6457	0.080*	
C27	0.5535 (2)	0.9385 (3)	0.71641 (16)	0.0662 (6)	
H27A	0.4904	0.9212	0.6677	0.079*	
H27B	0.5594	0.8698	0.7604	0.079*	
N1	0.89013 (12)	1.09912 (16)	0.42253 (11)	0.0472 (4)	
N2	0.49803 (12)	0.75259 (15)	0.40562 (9)	0.0403 (3)	
O1	0.5485 (2)	1.0718 (2)	0.75206 (12)	0.0920 (7)	
H1	0.5430	1.1294	0.7145	0.138*	
H5	0.8714 (18)	0.687 (3)	0.3262 (15)	0.054 (6)*	
H8	0.7093 (18)	0.729 (2)	0.4023 (15)	0.055 (6)*	
H11	0.7485 (17)	1.189 (3)	0.4958 (14)	0.054 (6)*	
H23	0.3214 (17)	0.525 (2)	0.5550 (15)	0.053 (6)*	
H2	1.061 (2)	1.098 (3)	0.3724 (15)	0.058 (7)*	
H4	1.025 (2)	0.702 (3)	0.2924 (18)	0.077 (8)*	
H10	0.621 (2)	1.054 (2)	0.5176 (15)	0.062 (7)*	
H17	0.4891 (16)	0.649 (2)	0.6239 (14)	0.046 (5)*	
H3	1.116 (2)	0.910 (3)	0.309 (2)	0.090 (9)*	
H20	0.342 (2)	0.653 (3)	0.2771 (19)	0.079 (8)*	
H21	0.196 (2)	0.546 (3)	0.2859 (17)	0.073 (8)*	

### Atomic displacement parameters (Å^2^)

**Table:**

	*U*^11^	*U*^22^	*U*^33^	*U*^12^	*U*^13^	*U*^23^
C1	0.0384 (9)	0.0591 (11)	0.0381 (9)	0.0007 (8)	0.0111 (7)	0.0064 (8)
C2	0.0450 (11)	0.0906 (17)	0.0447 (10)	−0.0091 (11)	0.0181 (9)	0.0069 (11)
C3	0.0557 (14)	0.110 (2)	0.0526 (12)	−0.0038 (13)	0.0286 (11)	−0.0064 (13)
C4	0.0649 (15)	0.0946 (19)	0.0524 (12)	0.0144 (14)	0.0292 (11)	−0.0067 (12)
C5	0.0568 (12)	0.0616 (13)	0.0482 (11)	0.0054 (10)	0.0218 (9)	−0.0026 (9)
C6	0.0396 (9)	0.0535 (10)	0.0372 (8)	0.0047 (7)	0.0125 (7)	0.0046 (7)
C7	0.0368 (9)	0.0408 (9)	0.0395 (8)	0.0040 (6)	0.0122 (7)	0.0028 (7)
C8	0.0416 (9)	0.0356 (8)	0.0447 (9)	0.0021 (7)	0.0156 (7)	0.0006 (7)
C9	0.0360 (8)	0.0386 (8)	0.0444 (9)	0.0018 (6)	0.0149 (7)	0.0024 (7)
C10	0.0396 (10)	0.0417 (9)	0.0580 (11)	0.0036 (7)	0.0204 (8)	−0.0029 (8)
C11	0.0442 (10)	0.0349 (9)	0.0627 (12)	0.0002 (7)	0.0188 (9)	−0.0043 (8)
C12	0.0366 (8)	0.0406 (9)	0.0456 (9)	−0.0004 (7)	0.0107 (7)	0.0053 (7)
C13	0.0564 (12)	0.0512 (11)	0.0601 (12)	−0.0159 (9)	0.0183 (10)	−0.0019 (9)
C14	0.0953 (19)	0.0568 (14)	0.0723 (16)	0.0008 (13)	0.0358 (14)	0.0139 (11)
C15	0.0368 (8)	0.0348 (8)	0.0409 (8)	0.0033 (6)	0.0157 (7)	−0.0005 (6)
C16	0.0386 (8)	0.0368 (8)	0.0386 (8)	0.0049 (6)	0.0122 (7)	−0.0020 (6)
C17	0.0455 (9)	0.0383 (8)	0.0358 (8)	0.0037 (7)	0.0166 (7)	0.0006 (6)
C18	0.0391 (8)	0.0316 (7)	0.0407 (8)	0.0025 (6)	0.0165 (7)	−0.0008 (6)
C19	0.0398 (9)	0.0379 (8)	0.0388 (8)	0.0007 (7)	0.0146 (7)	−0.0013 (6)
C20	0.0533 (11)	0.0585 (12)	0.0385 (9)	−0.0072 (9)	0.0101 (8)	−0.0015 (8)
C21	0.0496 (11)	0.0551 (12)	0.0531 (11)	−0.0079 (9)	0.0059 (9)	−0.0055 (9)
C22	0.0431 (10)	0.0370 (9)	0.0636 (12)	−0.0020 (7)	0.0178 (9)	−0.0029 (8)
C23	0.0456 (10)	0.0360 (8)	0.0536 (10)	0.0009 (7)	0.0222 (8)	0.0022 (7)
C24	0.0532 (13)	0.0551 (13)	0.0873 (17)	−0.0124 (10)	0.0214 (12)	−0.0005 (12)
C25	0.0516 (11)	0.0511 (11)	0.0438 (10)	−0.0017 (8)	0.0086 (8)	−0.0059 (8)
C26	0.0769 (16)	0.0637 (14)	0.0556 (13)	−0.0054 (12)	0.0160 (11)	−0.0107 (10)
C27	0.0820 (17)	0.0611 (14)	0.0537 (12)	0.0008 (12)	0.0203 (12)	0.0013 (10)
N1	0.0416 (8)	0.0478 (9)	0.0525 (9)	−0.0064 (6)	0.0163 (7)	0.0038 (7)
N2	0.0428 (8)	0.0422 (8)	0.0375 (7)	−0.0012 (6)	0.0155 (6)	0.0008 (6)
O1	0.161 (2)	0.0715 (12)	0.0560 (10)	0.0272 (13)	0.0528 (12)	−0.0011 (8)

### Geometric parameters (Å, °)

**Table:**

C1—N1	1.372 (3)	C15—N2	1.321 (2)
C1—C2	1.395 (3)	C15—C16	1.428 (2)
C1—C6	1.413 (3)	C16—C17	1.364 (3)
C2—C3	1.364 (4)	C16—C25	1.516 (2)
C2—H2	0.95 (2)	C17—C18	1.408 (2)
C3—C4	1.390 (4)	C17—H17	0.97 (2)
C3—H3	0.99 (3)	C18—C19	1.408 (2)
C4—C5	1.385 (4)	C18—C23	1.414 (2)
C4—H4	0.98 (3)	C19—N2	1.365 (2)
C5—C6	1.390 (3)	C19—C20	1.415 (3)
C5—H5	0.96 (2)	C20—C21	1.352 (3)
C6—C7	1.444 (3)	C20—H20	0.99 (3)
C7—C8	1.389 (3)	C21—C22	1.414 (3)
C7—C12	1.405 (3)	C21—H21	0.99 (3)
C8—C9	1.388 (3)	C22—C23	1.366 (3)
C8—H8	1.00 (2)	C22—C24	1.505 (3)
C9—C10	1.397 (3)	C23—H23	1.05 (2)
C9—C15	1.490 (2)	C24—H24A	0.96
C10—C11	1.373 (3)	C24—H24B	0.96
C10—H10	0.95 (3)	C24—H24C	0.96
C11—C12	1.382 (3)	C25—C26	1.518 (3)
C11—H11	0.96 (2)	C25—H25A	0.97
C12—N1	1.382 (2)	C25—H25B	0.97
C13—N1	1.442 (3)	C26—C27	1.483 (4)
C13—C14	1.501 (3)	C26—H26A	0.97
C13—H13A	0.97	C26—H26B	0.97
C13—H13B	0.97	C27—O1	1.448 (3)
C14—H14A	0.96	C27—H27A	0.97
C14—H14B	0.96	C27—H27B	0.97
C14—H14C	0.96	O1—H1	0.82
			
N1—C1—C2	129.3 (2)	C17—C16—C25	120.02 (17)
N1—C1—C6	109.41 (17)	C15—C16—C25	122.67 (17)
C2—C1—C6	121.3 (2)	C16—C17—C18	121.29 (16)
C3—C2—C1	117.8 (2)	C16—C17—H17	120.7 (13)
C3—C2—H2	123.9 (15)	C18—C17—H17	117.9 (13)
C1—C2—H2	118.2 (15)	C19—C18—C17	117.25 (16)
C2—C3—C4	122.1 (2)	C19—C18—C23	119.47 (16)
C2—C3—H3	118.3 (19)	C17—C18—C23	123.28 (16)
C4—C3—H3	119.6 (19)	N2—C19—C18	122.06 (15)
C5—C4—C3	120.5 (2)	N2—C19—C20	119.33 (16)
C5—C4—H4	119.5 (17)	C18—C19—C20	118.59 (17)
C3—C4—H4	119.7 (17)	C21—C20—C19	120.50 (19)
C4—C5—C6	118.9 (2)	C21—C20—H20	118.1 (16)
C4—C5—H5	119.3 (14)	C19—C20—H20	121.2 (16)
C6—C5—H5	121.7 (14)	C20—C21—C22	121.63 (19)
C5—C6—C1	119.39 (19)	C20—C21—H21	119.3 (16)
C5—C6—C7	134.31 (19)	C22—C21—H21	118.6 (16)
C1—C6—C7	106.28 (17)	C23—C22—C21	118.70 (18)
C8—C7—C12	119.11 (17)	C23—C22—C24	121.7 (2)
C8—C7—C6	134.49 (17)	C21—C22—C24	119.60 (19)
C12—C7—C6	106.33 (16)	C22—C23—C18	121.07 (18)
C9—C8—C7	119.75 (16)	C22—C23—H23	119.2 (13)
C9—C8—H8	119.4 (14)	C18—C23—H23	119.7 (13)
C7—C8—H8	120.8 (14)	C22—C24—H24A	109.5
C8—C9—C10	119.14 (17)	C22—C24—H24B	109.5
C8—C9—C15	119.86 (15)	H24A—C24—H24B	109.5
C10—C9—C15	120.91 (16)	C22—C24—H24C	109.5
C11—C10—C9	122.51 (18)	H24A—C24—H24C	109.5
C11—C10—H10	116.8 (15)	H24B—C24—H24C	109.5
C9—C10—H10	120.7 (15)	C16—C25—C26	117.85 (18)
C10—C11—C12	117.50 (17)	C16—C25—H25A	107.8
C10—C11—H11	122.1 (14)	C26—C25—H25A	107.8
C12—C11—H11	120.4 (14)	C16—C25—H25B	107.8
C11—C12—N1	128.64 (18)	C26—C25—H25B	107.8
C11—C12—C7	121.90 (17)	H25A—C25—H25B	107.2
N1—C12—C7	109.45 (17)	C27—C26—C25	115.1 (2)
N1—C13—C14	113.27 (19)	C27—C26—H26A	108.5
N1—C13—H13A	108.9	C25—C26—H26A	108.5
C14—C13—H13A	108.9	C27—C26—H26B	108.5
N1—C13—H13B	108.9	C25—C26—H26B	108.5
C14—C13—H13B	108.9	H26A—C26—H26B	107.5
H13A—C13—H13B	107.7	O1—C27—C26	109.2 (2)
C13—C14—H14A	109.5	O1—C27—H27A	109.8
C13—C14—H14B	109.5	C26—C27—H27A	109.8
H14A—C14—H14B	109.5	O1—C27—H27B	109.8
C13—C14—H14C	109.5	C26—C27—H27B	109.8
H14A—C14—H14C	109.5	H27A—C27—H27B	108.3
H14B—C14—H14C	109.5	C1—N1—C12	108.52 (16)
N2—C15—C16	123.25 (16)	C1—N1—C13	126.70 (18)
N2—C15—C9	114.92 (15)	C12—N1—C13	124.74 (18)
C16—C15—C9	121.83 (15)	C15—N2—C19	118.85 (15)
C17—C16—C15	117.26 (15)	C27—O1—H1	109.5
			
N1—C1—C2—C3	178.2 (2)	C15—C16—C17—C18	−2.1 (2)
C6—C1—C2—C3	−0.1 (3)	C25—C16—C17—C18	−179.67 (16)
C1—C2—C3—C4	1.0 (4)	C16—C17—C18—C19	2.3 (3)
C2—C3—C4—C5	−0.9 (4)	C16—C17—C18—C23	−177.00 (16)
C3—C4—C5—C6	0.0 (4)	C17—C18—C19—N2	−0.8 (3)
C4—C5—C6—C1	0.8 (3)	C23—C18—C19—N2	178.51 (16)
C4—C5—C6—C7	−176.9 (2)	C17—C18—C19—C20	−179.44 (17)
N1—C1—C6—C5	−179.39 (17)	C23—C18—C19—C20	−0.1 (3)
C2—C1—C6—C5	−0.8 (3)	N2—C19—C20—C21	−177.61 (19)
N1—C1—C6—C7	−1.1 (2)	C18—C19—C20—C21	1.0 (3)
C2—C1—C6—C7	177.49 (17)	C19—C20—C21—C22	−0.3 (3)
C5—C6—C7—C8	1.8 (4)	C20—C21—C22—C23	−1.4 (3)
C1—C6—C7—C8	−176.15 (19)	C20—C21—C22—C24	178.5 (2)
C5—C6—C7—C12	178.6 (2)	C21—C22—C23—C18	2.3 (3)
C1—C6—C7—C12	0.70 (19)	C24—C22—C23—C18	−177.59 (18)
C12—C7—C8—C9	−1.7 (3)	C19—C18—C23—C22	−1.6 (3)
C6—C7—C8—C9	174.82 (18)	C17—C18—C23—C22	177.69 (17)
C7—C8—C9—C10	3.4 (3)	C17—C16—C25—C26	−92.1 (2)
C7—C8—C9—C15	179.91 (15)	C15—C16—C25—C26	90.4 (2)
C8—C9—C10—C11	−2.1 (3)	C16—C25—C26—C27	61.1 (3)
C15—C9—C10—C11	−178.59 (18)	C25—C26—C27—O1	−177.84 (19)
C9—C10—C11—C12	−0.9 (3)	C2—C1—N1—C12	−177.38 (19)
C10—C11—C12—N1	−175.81 (18)	C6—C1—N1—C12	1.0 (2)
C10—C11—C12—C7	2.6 (3)	C2—C1—N1—C13	4.8 (3)
C8—C7—C12—C11	−1.3 (3)	C6—C1—N1—C13	−176.76 (18)
C6—C7—C12—C11	−178.78 (17)	C11—C12—N1—C1	177.99 (19)
C8—C7—C12—N1	177.34 (15)	C7—C12—N1—C1	−0.6 (2)
C6—C7—C12—N1	−0.09 (19)	C11—C12—N1—C13	−4.2 (3)
C8—C9—C15—N2	−62.5 (2)	C7—C12—N1—C13	177.27 (17)
C10—C9—C15—N2	113.96 (19)	C14—C13—N1—C1	95.3 (3)
C8—C9—C15—C16	116.72 (19)	C14—C13—N1—C12	−82.2 (3)
C10—C9—C15—C16	−66.8 (2)	C16—C15—N2—C19	1.1 (3)
N2—C15—C16—C17	0.3 (3)	C9—C15—N2—C19	−179.70 (15)
C9—C15—C16—C17	−178.81 (15)	C18—C19—N2—C15	−0.8 (3)
N2—C15—C16—C25	177.89 (16)	C20—C19—N2—C15	177.79 (17)
C9—C15—C16—C25	−1.3 (3)		

### Hydrogen-bond geometry (Å, °)

**Table:**

*D*—H···*A*	*D*—H	H···*A*	*D*···*A*	*D*—H···*A*
O1—H1···N2^i^	0.82	2.18	2.987 (2)	166
C17—H17···O1^ii^	0.97 (2)	2.38 (2)	3.334 (3)	171 (2)
C10—H10···Cg1^i^	0.96 (3)	2.76 (2)	3.559 (2)	141 (2)

Symmetry codes: (i) −*x*+1, −*y*+2, −*z*+1; (ii) −*x*+1, *y*−1/2, −*z*+3/2.
